# Molecular Mechanisms of Lithium Action: Switching the Light on Multiple Targets for Dementia Using Animal Models

**DOI:** 10.3389/fnmol.2018.00297

**Published:** 2018-08-28

**Authors:** Fiona Kerr, Ivana Bjedov, Oyinkan Sofola-Adesakin

**Affiliations:** ^1^Department of Life Sciences, School of Health & Life Sciences, Glasgow Caledonian University, Glasgow, United Kingdom; ^2^UCL Cancer Institute, University College London, London, United Kingdom; ^3^Sussex Neuroscience, School of Life Sciences, University of Sussex, Brighton, United Kingdom

**Keywords:** lithium, dementia, GSK-3, oxidative damage, neuro-inflammation, proteostasis, neurogenesis, synaptic maintenance

## Abstract

Lithium has long been used for the treatment of psychiatric disorders, due to its robust beneficial effect as a mood stabilizing drug. Lithium’s effectiveness for improving neurological function is therefore well-described, stimulating the investigation of its potential use in several neurodegenerative conditions including Alzheimer’s (AD), Parkinson’s (PD) and Huntington’s (HD) diseases. A narrow therapeutic window for these effects, however, has led to concerted efforts to understand the molecular mechanisms of lithium action in the brain, in order to develop more selective treatments that harness its neuroprotective potential whilst limiting contraindications. Animal models have proven pivotal in these studies, with lithium displaying advantageous effects on behavior across species, including worms (*C. elegans*), zebrafish (*Danio rerio*), fruit flies (*Drosophila melanogaster*) and rodents. Due to their susceptibility to genetic manipulation, functional genomic analyses in these model organisms have provided evidence for the main molecular determinants of lithium action, including inhibition of inositol monophosphatase (IMPA) and glycogen synthase kinase-3 (GSK-3). Accumulating pre-clinical evidence has indeed provided a basis for research into the therapeutic use of lithium for the treatment of dementia, an area of medical priority due to its increasing global impact and lack of disease-modifying drugs. Although lithium has been extensively described to prevent AD-associated amyloid and tau pathologies, this review article will focus on generic mechanisms by which lithium preserves neuronal function and improves memory in animal models of dementia. Of these, evidence from worms, flies and mice points to GSK-3 as the most robust mediator of lithium’s neuro-protective effect, but it’s interaction with downstream pathways, including Wnt/β-catenin, CREB/brain-derived neurotrophic factor (BDNF), nuclear factor (erythroid-derived 2)-like 2 (Nrf2) and toll-like receptor 4 (TLR4)/nuclear factor-κB (NFκB), have identified multiple targets for development of drugs which harness lithium’s neurogenic, cytoprotective, synaptic maintenance, anti-oxidant, anti-inflammatory and protein homeostasis properties, in addition to more potent and selective GSK-3 inhibitors. Lithium, therefore, has advantages as a multi-functional therapy to combat the complex molecular pathology of dementia. Animal studies will be vital, however, for comparative analyses to determine which of these defense mechanisms are most required to slow-down cognitive decline in dementia, and whether combination therapies can synergize systems to exploit lithium’s neuro-protective power while avoiding deleterious toxicity.

## Introduction

Lithium has well-described clinical benefits as a mood-stabilizer, and accumulating pre-clinical evidence has provided a basis for research into its therapeutic use in the treatment of a range of neurodegenerative conditions, including Alzheimer’s (AD), Parkinson’s (PD) and Huntington’s (HD) diseases (De Ferrari et al., 2003; Noble et al., 2005; Sarkar et al., 2008; Chiu et al., 2011; Lieu et al., 2014). A narrow therapeutic window for these effects, however, has led to concerted efforts to understand the molecular mechanisms of lithium action in the central nervous system (CNS), in order to develop more selective treatments that harness its neuroprotective potential whilst limiting its toxic side-effects.

Animal models have proven pivotal in these studies, with lithium displaying advantageous effects on behavior across species, including worms (*Caenorhabditis elegans*; Farina et al., 2017), zebrafish (*Danio Rerio*; Nery et al., 2014), fruit flies (*Drosophila melanogaster*; Mudher et al., 2004; McBride et al., 2005; Sofola et al., 2010; Castillo-Quan et al., 2016) and rodents (O’Brien et al., 2004; King and Jope, 2013; Lu et al., 2015; Gelfo et al., 2017). Due to their susceptibility to genetic manipulation, these model organisms have provided evidence for the direct molecular determinants to which lithium’s neuro-protective effects are attributed, including inhibition of inositol monophosphatase (IMPA), glycogen synthase kinase-3 (GSK-3), and a plethora of down-stream targets that further exert neuroprotective potential. Lithium has an important role in cytoprotection particularly by preventing oxidative and neuro-inflammatory damage, maintaining protein homeostasis and enhancing neurogenesis and synaptic maintenance. These neuro-protective properties have beneficial effects across a range of animal models of neurodegeneration, and understanding the mechanisms underpinning these interactions has revealed new targets for the development of drugs to slow-down neuronal damage in these conditions.

Focussing on animal models of dementia, here we review the evidence for the molecular basis of the generic mechanisms by which lithium preserves neuronal function and improves memory. Animal studies will be vital, however, for comparative analyses to determine which of these defense mechanisms are most required to slow-down cognitive decline as disease progresses. We discuss the advantages of combination therapies to synergize the targets of lithium’s protective effects, in comparison with using lithium as a single multi-functional therapy to combat the complex molecular pathology of dementia.

## Multi-Faceted Mechanisms of Lithium-Mediated Neuroprotection: Evidence From Model Organisms

**Figure 1:**
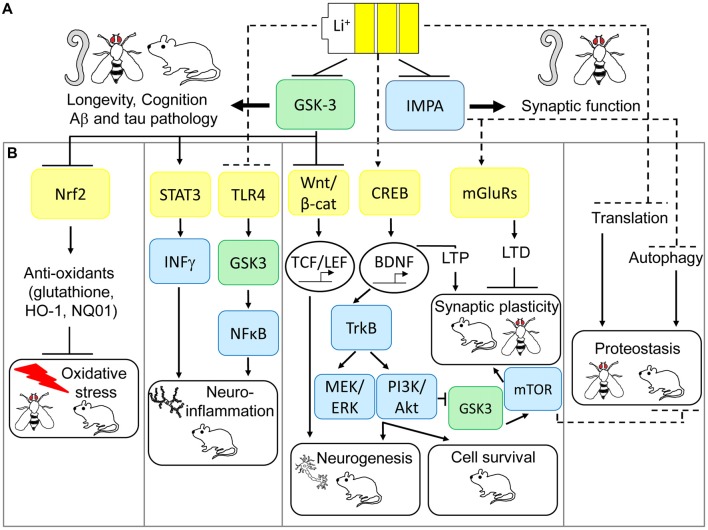
Multi-modal mechanisms of lithium-mediated neuronal protection in model organisms. **(A)** Glycogen synthase kinase-3 (GSK-3) and inositol monophosphatase (IMPA) are direct targets of lithium action in the central nervous system (CNS). Model organisms have revealed IMPA as a mediator of improved synaptic function in response to lithium, but IMPA1/2 mutations fail to consistently pheno-copy lithium’s protective effects on behavior in mice. GSK-3 appears to be a conserved mediator of lithium action, required for increased longevity, improved cognition and prevention of Alzheimer’s disease (AD) pathology across worm, fly and mice models of neurodegeneration. **(B)** Animal models have also uncovered several pathways which may preserve downstream neuro-protection processes in response to lithium via GSK-3-dependent and independent mechanisms. GSK-3 inhibition is an upstream regulator of lithium’s activation of nuclear factor (erythroid-derived 2)-like 2 (Nrf2) in preventing oxidative damage, inhibition of signal transducer and activator of transcription 3 (STAT3) to prevent neuro-inflammation, increased Wnt-dependent gene transcription to guide adult neurogenesis, and potentially prevention of protein synthesis by inhibiting translation. More recent evidence also suggests that lithium can exert neuro-protection through non-GSK-3-dependent anti-inflammatory effects on toll-like receptor 4 (TLR4), increased neurogenesis, cell survival and long term potentiation (LTP) via CREB-dependent transcription of brain-derived neurotrophic factor (BDNF) and prevention of long term depression (LTD) by inhibition of over-active metabotropic glutamate receptor (mGLuR)-dependent synaptic transmission. Finally, inhibition of IMPA mediates lithium-dependent activation of autophagy, by reducing inositol levels, thus maintaining protein turnover. Genetic and pharmacological modulation of these anti-oxidant, anti-inflammatory, neurogenesis, cell survival, synaptic plasticity and proteostasis signaling pathways prevents neurodegeneration and improves cognition in *Drosophila* and mouse models of AD, fronto-temporal dementia (FTD) and Fragile X syndrome.

Although the precise molecular determinants of lithium action remain unclear, evidence from model organisms have suggested that IMPA and GSK-3 are direct regulators of its beneficial effects on neuronal function (see Figure 1A). As originally posited by Berridge et al. (1989), the inositol depletion hypothesis of lithium action suggests that uncompetitive inhibition of IMPA and inositol polyphosphate 1-phosphatase (IPP) leads to a deficit in polyphosphoinositide (PIP) signaling, and subsequently inhibition of neuronal excitation, which may explain its beneficial effects as a mood stabilizer. Genetic mutation of IPP in *Drosophila melanogaster* (Acharya et al., 1998) or the IMPA homolog, ttx-7, in *C. elegans* (Tanizawa et al., 2006) has indeed shown that these enzymes play an important role in regulation of synaptic function *in vivo*, although this appears to be mediated by enhancing rather than suppressing synaptic transmission. Contrasting studies in mice, however, suggest that inositol depletion may not alter PIP levels in the CNS (Berry et al., 2004). Furthermore, IMPA1 deletion only partially mimics lithium’s effects on gene expression in the hippocampus (Damri et al., 2015) and IMPA2 deletion fails to phenocopy lithium’s protective effects against depression and anxiety-like behavior (Cryns et al., 2007). Hence the role of IMPA in mediating lithium’s function in the mammalian CNS is less clear.

GSK-3 is also non-competitively inhibited by lithium (Klein and Melton, 1996) and appears to be a more consistent regulator of lithium’s neuro-protective effects across species (Gurvich and Klein, 2002; Aghdam and Barger, 2007). GSK-3 is epistatically required for longevity in response to lithium in both *C. elegans* (McColl et al., 2008) and *Drosophila* (Castillo-Quan et al., 2016), and it’s mutation mimics lithium’s ability to alter exploratory behavior in mice (O’Brien et al., 2004, 2011). GSK-3 also plays a well-described role in the generation of AD-associated amyloid β (Aβ) and tau pathologies (Kremer et al., 2011), by promoting abnormal tau phosphorylation (Lucas et al., 2001) and increasing amyloid production (Phiel et al., 2003) and toxicity (DaRocha-Souto et al., 2012). Furthermore, GSK-3 inhibition protects against neuronal damage and cognitive decline in *Drosophila* (Mudher et al., 2004; Sofola et al., 2010) and rodent models of AD and fronto-temporal dementia (FTD; Serenó et al., 2009). As a pleiotropic enzyme, however, GSK-3 inhibition may exert its neuroprotective effects through diverse mechanisms, including maintenance of axonal transport and synaptic function, promoting adult neurogenesis, preventing apoptosis and reducing neuro-inflammation (Llorens-Martín et al., 2014). Building upon these observations, and as extensively reviewed (Eldar-Finkelman and Martinez, 2011), drug development efforts have aimed to discover new specific GSK-3 inhibitors with improved toxicity profiles for treatment of neurodegenerative disorders in comparison with lithium. These include ATP-competitive (indirubin, paullones, thiazoles, SB-216763 and SB-415286) and non-ATP-competitive (thiadiazolidindiones (TDZD-8, NP-12/tideglusib), and L803-mts) inhibitors, which have shown promising pre-clinical efficacy in improving cognition and protecting neurons in rodent models of AD and FTD. Of these, non-ATP-competitive GSK-3 inhibitors appear to have improved specificity and safety, which has led to Phase II clinical trials for the use of tideglusib in mild-moderate AD, as part of the ARGO study (Lovestone et al., 2015), and in progressive supranuclear palsy (PSP), as part of the TAUROS study (Tolosa et al., 2014). Although tideglusib proved safe for use in both AD and PSP patients, and some reduction in brain atrophy was observed in the TAUROS study, no significant clinical improvement in primary measures, ADAS-cog15 score or PSP rating scale, was reported in either of these trials. Based on the observation that mild AD patients in the ARGO study showed some cognitive improvement on lower doses of lithium (500 mg; Lovestone et al., 2015), suggesting a non-linear dose-response, future studies to examine the effects of tideglusib in patients at earlier stages and to optimize the most effective dose may be warranted.

Recent transcriptomic analyses across species, however, have revealed that lithium has wide-ranging cellular effects. For example by altering DNA replication, metabolism and endoplasmic reticulum (ER) genes in worms (McColl et al., 2008), translation and cellular detoxification genes in flies (Castillo-Quan et al., 2016), and neurogenesis, synaptic function, anti-apoptosis and anti-inflammatory genes in rat and mouse brain (Roux and Dosseto, 2017). This supports a multi-faceted mechanism of lithium-mediated neuroprotection, of relevance to many neurological conditions, through promotion of its cytoprotective, anti-oxidant, anti-inflammatory, protein homeostasis, neurogenic and synaptic maintenance properties. Alterations in these pathways may be an indirect consequence of GSK-3’s pleiotropic effects, but understanding the molecular basis of their modulation has revealed new selective targets for neuronal protection in dementia as we discuss below.

## Oxidative Stress: Nrf2 as a Mediator of Lithium’S Anti-oxidant Neuro-Protective Effects

Oxidative damage is a common feature of dementia brain (Sultana and Butterfield, 2010; Iadecola, 2013), with reactive oxygen species (ROS) and peroxidized lipids and proteins accumulating early in the disease process (Sultana and Butterfield, 2010). This may be a result of aging related mitochondrial damage, hypoxia-induced ischemia or amyloid accumulation, and leads to wide-spread cellular damage through prevention of cytoprotective signaling, induction of apoptosis and neuro-inflammation (Guo et al., 2017).

Lithium prevents neuronal sensitivity to oxidative damage across species, including hyperoxia in Aβ-expressing flies (Kerr et al., 2017), cerebral ischemia and 3-nitropropionic acid (3-NP)-induced neurotoxicity in rats (Khan et al., 2015; Chen et al., 2016), and kainate and neuropeptide S-induced behavioral and neurological damage in mice (Rojo et al., 2008; Castro et al., 2009). Prevention of oxidative damage by lithium in these paradigms commonly correlates with reduced lipid peroxidation, as measured by thiobarbituric acid reactive substances (TBARS; Shao et al., 2005; Castro et al., 2009) or 4-hydroxynonenal (4-HNE) levels (Tan et al., 2012), protein carbonylation (Shao et al., 2005) and ROS production (Rojo et al., 2008). Conversely lithium treatment increases expression of anti-oxidant enzymes including catalase (Khan et al., 2015), heme-oxygenase-1 (HO-1; Khan et al., 2015; Chen et al., 2016) and NAD(P)H: quinone oxidoreductase 1 (NQ01; Chen et al., 2016) as well as restoring levels of glutathione and glutathione-s-transferases (GstD1, GstD2), an important mediator of neuronal protection against oxidative damage (Baxter and Hardingham, 2016), in *Drosophila* (Kasuya et al., 2009; Kerr et al., 2017) and rat (Khan et al., 2015) brain. Notably, the HO-1 inhibitor SnPP reversed the beneficial effects of lithium against 3-NP-induced oxidative stress and motor defects in rats (Khan et al., 2015), providing epistatic evidence that prevention of oxidative damage plays a key role in lithium-mediated neuronal protection *in vivo*.

GSK-3 inhibition parallels many of these anti-oxidant properties of lithium, with GSK-3 antisense oligonucleotides, short-interfering RNAs (siRNA) and specific GSK-3 inhibitors (SB216763, TDZD-8; Rojo et al., 2008; Chen et al., 2016) preventing oxidative damage in rat models of cerebral ischemia and the SAMP8 mouse model of aging-related AD (Farr et al., 2014; Chen et al., 2016). The transcription factor nuclear factor (erythroid-derived 2)-like 2 (Nrf2) has been proposed as a key molecular mediator of the antioxidant effects of lithium down-stream of GSK-3 as reviewed by Kanninen et al. (2011; see Figure 1B). Nrf2 responds to cellular stress, for example under oxidative or neuro-inflammatory conditions, which are common features of neurodegenerative diseases, by increasing expression of an array of antioxidant response element (ARE)-containing genes to counteract damage (Bruns et al., 2015). Lithium increases Nrf2 activity in *Drosophila* (Castillo-Quan et al., 2016; Kerr et al., 2017), and pheno-copies specific GSK3β inhibition (siRNA and SB216763, TDZD-8) in increasing Nrf2 nuclear translocation and activating transcription of Nrf2 target genes, HO-1 and NQ01, in rat and mouse brain (Rojo et al., 2008; Chen et al., 2016). Moreover, Nrf2 is genetically required for lifespan extension by lithium in flies (Castillo-Quan et al., 2016) and increased Nrf2 nuclear translocation correlates with reduced oxidative damage and improved cognition in SAMP8 mice following treatment with antisense GSK-3 (Farr et al., 2014). These further suggest a causal role for Nrf2 in mediating the neuroprotective effects of lithium and GSK-3 inhibitors in dementia models.

As a general regulator of neuronal protection, Nrf2 has become an attractive therapeutic target for the treatment of several neurodegenerative diseases including AD (Kanninen et al., 2009) and vascular dementia (Alfieri et al., 2011). Genetic activation of Nrf2 protects against neuronal and cognitive decline in *Drosophila* and mouse models of AD and PD (Kanninen et al., 2009; Barone et al., 2011; Kerr et al., 2017), and pharmacological Nrf2 activators, including triterpenoid compounds (CDDO-EA and CDDO-TFEA) and dimethyl fumarate (DMF), prevent oxidative damage and afford neuronal protection in mouse models of AD (Dumont et al., 2009), FTD (Cuadrado et al., 2018), amyotrophic lateral sclerosis (ALS; Neymotin et al., 2011), PD (Chen et al., 2009; Lastres-Becker et al., 2016) and cerebral ischemia (Fowler et al., 2017). Most classical Nrf2 activators are electrophilic agents which modify cysteine residues on Keap1, a negative regulator of Nrf2, disrupting their physical interaction and enabling Nrf2 to activate transcription. The non-selective nature of this mechanism, however, is thought to explain their toxicity, due to off-target effects, as observed in clinical trials (Abed et al., 2015). Recent drug discovery efforts have therefore focussed on developing direct disruptors of the Keap1-Nrf2 protein-protein interaction (PPI), with the aim of improving safety profiles (Abed et al., 2015; Wells, 2015). We and others have indeed reported promising results using small molecule and peptide Keap1-Nrf2 PPI disruptors in protecting against amyloid-induced synapto-toxicity in mouse primary neurons (Kerr et al., 2017) and neuronal protection *in vivo* using rat models of global cerebral ischemeia (Tu et al., 2015). Of note, our own studies using *Drosophila* and neuroblastoma cells suggest that lithium and GSK-3 inhibitors (TDZD8) are weak activators of Nrf2, relative to Keap1-Nrf2 disruption, in response to amyloid toxicity (Kerr et al., 2017). Combined GSK-3 inhibitor and Nrf2 activator therapies may be an advantage, as additive protective effects against Aβ toxicity are observed with Keap1 mutation and lithium treatment in the fly (Kerr et al., 2017) and DMF has recently been shown to prevent tau phosphorylation through inhibition of GSK-3 and neuronal damage by subsequent activation of Nrf2 through Keap1 dependent and independent mechanisms (Cuadrado et al., 2018). Hence further work is required to establish the relative importance of these pathways in maintaining Nrf2 activity in AD and other neurodegenerative diseases.

## Neuro-Inflammation: TLR4/NFκB and STAT3 as Anti-inflammatory Mediators of Lithium-Mediated Neuroprotection

Alterations in microglia activation and inflammatory factors are an early feature in the brains of dementia patients, including AD and vascular dementia (Calsolaro and Edison, 2016). Moreover, neuronal inflammation directly correlates with decline in cognitive function across species, for example following bacterial infection in flies (Cao et al., 2013; Wu et al., 2017) and viral infection (Hosseini et al., 2018) in mice, suggesting a causal link between inflammatory dysregulation and memory impairment. Based on the increasing risk associated with immunity genes (including TREM2 and CD33) and early neuropathological detection of inflammation in late-onset AD (LOAD), emerging theories also suggest that chronic, aging-associated, inflammation may be an initiating factor leading to the disease-defining neuronal loss, amyloid and tau pathologies of Alzheimer’s (as reviewed in Nazem et al., 2015). Amyloid aggregates may then induce further microglial dysfunction exacerbating a vicious cycle of neuro-inflammation and progression of disease. Several rodent models of AD have been developed based on this inflammation hypothesis, including peripheral immune challenge with bacterial lipopolysaccharide (LPS; Pintado et al., 2012) or viral polyriboinosinic-polyribocytidilic acid (PolyI:C) proteins (Krstic et al., 2012), intracerebroventricular (i.c.v) injection of streptozotocin (STZ; Chen et al., 2013) and transgenic over-expression of p25 (Sundaram et al., 2012), resulting in neuro-inflammation, amyloid plaques, NFTs and cognitive dysfunction. Moreover, mice and rat models of traumatic brain injury (Yu et al., 2012) and cerebral ischemia (Kawabori and Yenari, 2015) result in neuro-inflammation, including increase in toll-like receptors (TLRs), microglial activation, and expression of pro-inflammatory enzymes (cyclo-oxygenase-2, COX-2) and cytokines (IL-1β, TNFα), in correlation with secondary cognitive defects comparable to TBI (Vincent et al., 2014) and vascular-related dementias (Kawabori and Yenari, 2015).

Alterations in such neuro-inflammatory factors have indeed been shown to correlate with cognitive improvement by lithium using these mammalian models. Studies using a rat LPS model of neuro-inflammation identified lithium as an anti-inflammatory agent through its prevention of neuro-inflammatory prostaglandin production, for example PGE_2_ and TXB_2_ (Basselin et al., 2007), and elevation of the anti-inflammatory docosanoid 17*S*-hydroxy-DHA (17-OH-DHA; Basselin et al., 2010). Consistent with these observations, lithium treatment also prevented traumatic brain injury and cerebral ischemia-induced microglial activation, in mouse and rat brain, and this associated with prevention of neuronal loss (Li et al., 2011; Yu et al., 2012) and anxiety-like behavior using an open-field test (Yu et al., 2012). Correlating with prevention of cognitive defects, lithium carbonate improved spatial reference and working memory in a rat intra-hippocampal injection model of AD and reversed reductions in anti-inflammatory (IL-4) and increases in pro-inflammatory (IL-1β, TNFα) cytokines in the frontal cortex and hippocampus (Budni et al., 2017).

The molecular mediators of lithium’s anti-inflammatory effects in dementia models are unclear, but correlative evidence suggest that GSK-3, toll-like receptor 4 (TLR4), signal transducer and activator of transcription (STAT) and nuclear factor-κB (NFκB) pathways may play a causal role (Jope et al., 2017; see Figure 1B). Pro-inflammatory agents such as LPS and 6-hydroxydopamine (6-OHDA) increase microglial GSK-3 activity *in vitro* (Yuskaitis and Jope, 2009; Green and Nolan, 2012). Conversely, both lithium and specific GSK-3 inhibitors prevented neuro-inflammatory IL-1β, IL-6, TNFα and nitric oxide (NO) production in rat microglia (Yuskaitis and Jope, 2009; Green and Nolan, 2012) and astrocytes (Wang et al., 2013), as well as in mouse hippocampal slice cultures in correlation with neuronal protection (Yuskaitis and Jope, 2009). Alterations in expression of TLR4 and its target transcription factor NFκB, a key regulatory pathway in microglial activation and cytokine production, correlated with lithium’s rescue of surgery-induced memory impairment in aged rats in association with reductions in hippocampal TNF-α and IL-1β (Lu et al., 2015). Additionally, using TLR4 knockout mice, Cheng et al. (2016) demonstrated that TLR4 was required for anxiety-induced GSK-3 activation in the hippocampus and subsequently that GSK-3 inhibition, using TDZD-8, alleviated increased cytokine and chemokine production and NFκB activation in this model. Alternatively, GSK-3 phosphorylates and activates STAT3 by promoting its association with the pro-inflammatory interferon-γ (IFN-γ) receptor in mouse primary astrocytes, by mechanisms which appear independent of TLR4; and lithium and TDZD-8 block these effects (Beurel and Jope, 2008). Together these data suggest that GSK-3 may mediate lithium’s anti-inflammatory and neuro-protective effects by independently modulating TLR4/NFκB and STAT3 signaling. However, further research is required to delineate these pathways by measuring the effects of altering TLR4, NFκB and STAT3 on lithium and GSK-3 inhibitor-mediated neuronal protection in animal models.

Although lithium’s prevention of neuro-inflammation may simply represent one of the multi-faceted mechanisms by which GSK-3 inhibition protects neurons, the above evidence also suggests that TLR4 is a direct target for lithium, upstream of GSK-3 (see Figure 1B). Genetic modification of TLR4 protected against anxiety-like behavior in mice (Cheng et al., 2016), as well as Aβ and oxidative stress-induced toxicity in primary mouse neurons (Tang et al., 2008). Conversely Aβ and 4-HNE increased TLR4 protein in mouse neurons, and TLR4 levels were altered in AD patient brain (Tang et al., 2008), further suggesting that this may represent an attractive therapeutic target for AD. Consistently, the naturally-occurring compound gx-50 prevented Aβ-induced microglial activation and neuro-inflammation (IL-1β, TNFα, NO, COX2) in rat microglia cultures and APP transgenic mouse brain, to levels comparable with TLR4 gene silencing, and this correlated with a rescue of Aβ-induced enhancement of TLR4 protein *in vitro* and *in vivo* (Shi et al., 2016). Moreover, small molecule antagonists of TLR4 have recently been developed (De Paola et al., 2016) and protect against neuro-toxicity using *in vitro* models of ALS (De Paola et al., 2016) and LPS-induced neuro-inflammation (Perrin-Cocon et al., 2017), suggesting that an investigation of the potential of these drugs in dementia models is warranted. Finally, targeted modulation of NFκB and STAT3 pathways may offer selective protection against neuro-inflammation in dementia, for example using natural and rationally-designed inhibitors of the NFκB pathway (as reviewed, Srinivasan and Lahiri, 2015) and small molecule STAT3 inhibitors which are effective in cancer models (Fletcher et al., 2011). This requires empirical comparison with the efficacy of lithium and GSK-3 inhibitors against neuronal protection using *in vitro* and *in vivo* models of AD and other dementias.

## Neuronal Proteostasis: Lithium Maintains Protein Turnover by Blocking Translation and Enhancing Autophagy

Protein homeostasis is maintained by balancing protein synthesis and degradation. Qualitative adaptation of the proteome, as well as the rapid removal of damaged and unfolded proteins, is essential for normal cell functioning and this is particularly important for non-diving cells, such as neurons, where damaged components cannot be excluded through cell division (Douglas and Dillin, 2010; Balchin et al., 2016). Interestingly, lithium impacts on several aspects of proteostasis, for example by reducing protein synthesis and increasing protein degradation through modulation of proteasomal activity and induction of autophagy. This may prove beneficial for many neurodegenerative diseases, including AD and other forms of dementia, either by enhancing clearance of abnormal protein aggregates, which are a common feature of these conditions, or by maintaining protein homeostasis and therefore preserving neuronal cell function.

Initial evidence for the effect of lithium on reduction of protein synthesis came from studies on the development of sea urchin embryos (Berg, 1968). Lithium treatment using clinically-relevant lithium doses (0.6–1.0 mM) was able to down-regulate the expression of the epsilon subunit of the initiation factor-2B (eIF2B) in rat brain suggesting down-regulation of protein synthesis (Bosetti et al., 2002). Interestingly, *in vitro* experiments using the SH-SY5Y human neuroblastoma cell line showed that lithium had a positive effect on translation through reduced inhibitory phosphorylation of elongation factor eEF2, while GSK-3 inhibitors had opposing effects on phospho-eEF2, thereby reducing translation (Karyo et al., 2010). Similarly, GSK-3 was a positive regulator of translation in breast cancer cells, via phosphorylation of 4E-BP1 and a concomitant increase in eIF4E-dependent protein synthesis (Shin et al., 2014). Using a different *in vitro* system, CHO.T cells, it was shown that GSK-3 negatively affects translation by mediating inhibitory phosphorylation of eIF2B (Welsh et al., 1998). These conflicting results may depend on the system studied and differences between *in vitro* and *in vivo* approaches, but lithium consistently reduces protein synthesis across species in models of neurological diseases. Low lithium concentrations decreased levels of cerebral protein synthesis to control levels in the *Fmr1* KO mouse model of Fragile X syndrome, but had no effect on translation in wild-type mice (Liu et al., 2012). Lithium reduced protein synthesis, accompanied by increased longevity, in *Drosophila* models of AD (Sofola et al., 2010; Sofola-Adesakin et al., 2014), and experiments in yeast (*Schizosaccharomyces pombe*) confirmed the inhibitory effect of lithium on translation and its anti-aging effect as measured by chronological lifespan (Sofola-Adesakin et al., 2014). Although the precise molecular mechanisms remain unclear, several *in vivo* approaches therefore demonstrate reduced protein synthesis in response to lithium (Figure 1B). The benefits of this may include improved neuronal function by re-investing energy in cell maintenance processes, or delaying formation of protein aggregates by improving protein quality due to the increased availability of the protein folding and degradation machinery.

Lithium treatment is also increasingly linked to degradation pathways such as autophagy. Autophagy is a process whereby a portion of the cytoplasm is packaged into autophagosomes and subsequently, upon fusion with the lysosome, the content is degraded (Mizushima and Komatsu, 2011; Nikoletopoulou et al., 2015; Suzuki et al., 2017). Autophagy can degrade selective or random cargo and it is the only process that can degrade entire organelles (Mizushima and Komatsu, 2011). The main autophagy inducers are starvation and lack of growth factors which, through inhibition of mammalian target of rapamycin (mTOR) signaling, then trigger ULK1 complex (ULK1-ATG13-FIP200-ATG101) formation at Atg9-containing membranes and activation of the class 3 phosphatidylinositol-3-OH kinase (PI(3)K) complex (Jung et al., 2010). Significantly, autophagy can degrade protein aggregates and its reduction and defects have been consistently considered as contributing factors towards neurodegeneration (Tanaka and Matsuda, 2014; Frake et al., 2015). Moreover, knock-out mice that lack ATG5 or ATG7 autophagy genes in neurons, although viable, suffer from motor and behavioral defects, correlating with ubiquitinated protein aggregation and apoptosis (Hara et al., 2006; Komatsu et al., 2006). This suggests that insufficient autophagic clearance may directly cause neurodegeneration. Compounds that enhance autophagy, such as lithium, are therefore a promising therapeutic strategy for a variety of neurodegenerative conditions, such as AD, FTD, PD, HD and ALS, by removing toxic protein aggregates and/or improving mitochondrial health via mitophagy (Frake et al., 2015; Lionaki et al., 2015; Galluzzi et al., 2017; Menzies et al., 2017). For example, lithium treatment ameliorated motor dysfunction in mice overexpressing FTD-mutated human tau (P301L) via increased autophagy (Shimada et al., 2012). Low dose lithium also delayed disease in ALS patients (Fornai et al., 2008a), and neuroprotection by lithium in the SOD1G93A mouse model of ALS has been attributed to increased autophagy (Fornai et al., 2008a,b). Although some studies report no prevention of disease progression in this model (Pizzasegola et al., 2009). Numerous studies therefore report that lithium treatment is beneficial for a variety of neurodegenerative disorders owing to its capacity to enhance autophagy (Motoi et al., 2014). It is important to note, however, that there are exceptions where improvements in memory and decreased amyloid plaque formation following lithium treatment of mice expressing APPswe/PS1A246E associated with decreased autophagy (Zhang et al., 2011). These discrepancies may be explained by methodological differences in measuring autophagy or by variations in the lithium concentration used (Klionsky et al., 2016).

Interestingly, lithium has been shown to induce authophagy through mTOR-independent mechanisms (Figure 1B), more specifically via inhibition of IMPA leading to free inositol depletion and reduced myo-inositol-1,4,5-triphosphate (IP3) levels (Sarkar et al., 2005; Sarkar and Rubinsztein, 2006). This effect is replicated by the IMPA inhibitor L-690,330 (Atack et al., 1993) which enhanced the degradation of A53T α-synuclein and HDQ74 mutant huntingtin protein-expressing PC12 cells (Sarkar et al., 2005). GSK3β inhibition using SB216763 did not have any effect on autophagy induction or mutant HDQ74 protein clearance (Sarkar et al., 2005), providing further support that the effects of lithium on autophagy are GSK-3 independent. GSK3β inhibition can, in fact, have opposing effects and reduce autophagy through activation of the mTOR pathway. As these two lithium targets, IMPA and GSK3β, have independent but opposing effects on autophagy, combination of lithium with the mTOR inhibitor rapamycin, which has beneficial neuroprotective effects in *Drosophila* and mice models of AD and FTD (Khurana et al., 2006; Spilman et al., 2010; Ozcelik et al., 2013; Lin et al., 2017), maximized autophagy induction and clearance of mutant aggregate prone proteins more effectively than singular treatments using *in vitro* and *Drosophila* model of HD (Sarkar et al., 2008).

## Cross-Talk Between Wnt, BDNF and mGluR Signaling Pathways Mediates Lithium-Dependent Neurogenesis, Cell Survival and Synaptic Plasticity

Lithium plays many crucial roles required for proper nervous system function, such as promoting neurogenesis, synaptic plasticity and cell survival (Chen et al., 2000; Schloesser et al., 2008; Kim and Thayer, 2009; see Figure 1B). Neurogenesis is vital for hippocampal plasticity, and persists into adulthood in the mammalian brain. It underlies learning and memory processes, and thus alterations in neurogenesis are implicated in neurodegenerative diseases such as AD (Taupin, 2008). Several animal studies have investigated neurogenesis in AD with rather conflicting results. For example, two studies that utilized APP_Swe, Ind_ transgenic mice, expressing both the Swedish and Indiana amyloid precursor protein (APP) mutations but under different promoters, reported opposing phenotypic outcomes. One study reported an enhancement in neurogenesis in the hippocampus of transgenic mice relative to controls (Jin et al., 2004), while the other showed a reduction (Fiorentini et al., 2010). In the latter study, however, treatment of young mice with lithium, at 3 months of age, significantly increased neurogenesis in correlation with activation of Wnt signaling, as measured by increased nuclear β-catenin staining in newborn neurons (Fiorentini et al., 2010). Canonical Wnt signaling inhibits GSK3, thus enabling translocation of β-catenin to the nucleus and enhancing its association with the TCF/LEF family of transcription factors. Subsequent transcription of Wnt/β-catenin target genes leads to regulation of diverse processes that are critical for development of the mammalian CNS such as synapse plasticity and neurogenesis (Valvezan and Klein, 2012). Similarly, lithium pheno-copies the effects of Wnt signaling on GSK-3 inhibition and modulates neurogenesis and synaptic plasticity via activation of downstream β-catenin/TCF-LEF target genes (Fiorentini et al., 2010; Valvezan and Klein, 2012; Morris and Berk, 2016). Moreover, lithium is able to suppress astrogliogenesis (generation of new astroglia/astrocytes), through non-GSK-3-mediated mechanisms. Hence lithium may increase the neuronal fate of neuronal stem cells in two ways, by increasing neurogenesis and reducing astrogliogenesis (Zhu et al., 2011).

Cell survival by lithium is mediated by upregulation of basal adenylate cyclase activity, and thereby cyclic AMP (cAMP) and protein kinase A (PKA), which leads to CREB-dependent transcription of brain-derived neurotrophic factor (BDNF) and anti-apoptotic B cell lymphoma protein-2 (Bcl-2) genes (Chen and Chuang, 1999; Quiroz et al., 2010). Bcl-2 has been attributed to lithium’s anti-apoptotic effects, potentially by decreasing the pro-apoptotic Bax and p53 genes (Sugawara et al., 2010; Can et al., 2014). Bax promotes mitochondrial cytochrome *c* release subsequently promoting cytosolic activation of caspases and thus the degradation of specific protein substrates (Chen and Chuang, 1999). Bcl-2 counteracts these effects by preventing cytochrome *c* translocation and thus inhibiting Bax-induced caspase activation (Kluck et al., 1997). Alterations in the expression of Bcl-2 family members in AD brain have indicated a role for apoptosis in neuronal loss in this condition, but the role of caspase activation in mediating effects on AD pathology are unclear. Over-expression of Bcl-2 or neutralizing antibodies for Bax, however, prevented Aβ-induced death *in vitro* using primary neurons (Kudo et al., 2012). Lithium-mediated GSK-3 inhibition suppresses apoptosis (Chin et al., 2005), but it should be noted that the specific role of bcl-2 in mediating this effect is unclear as recent data has challenged alterations in its expression in response to lithium in light of optimal normalizing probes (Odeya et al., 2018).

Neurotrophins are also important mediators of cell survival, neurogenesis and synaptic maintenance. BDNF binds to tyrosine receptor kinase B (TrkB), both of which are widely expressed in the developing and adult mammalian brain, and activates phospholipase Cγ (PLCγ), phosphatidylinositol 3-kinase (PI3K), and extracellular signal-regulated kinase (ERK) pathways which are involved in neuronal differentiation, preventing apoptosis, promoting cell survival and maintaining synaptic plasticity (Tajes et al., 2009; Cunha et al., 2010). BDNF/TrkB-induced PI3K signaling leads to phosphorylation and inhibition of GSK-3, enabling the activation of downstream effectors, including mTOR, that play a key role in synaptic remodeling (Cunha et al., 2010; Morris and Berk, 2016). Moreover, BDNF plays an important role in the induction and maintenance of long term potentiation (LTP), synaptic transmission by acting at pre- and postsynaptic sites, and presynaptic release of the excitatory neurotransmitter glutamate (Jovanovic et al., 2000; Wurzelmann et al., 2017). Patients with bipolar disorder have significantly decreased BDNF in blood serum levels compared to healthy controls (Fernandes et al., 2015), and conversely lithium treatment is associated with increased serum BDNF protein in these patients (Gideons et al., 2017). Chronic lithium treatment has been shown to increase BDNF mRNA and protein expression across brain regions in rodent and mice models (Fukumoto et al., 2001; Hashimoto et al., 2002; Gideons et al., 2017), as well as in cortical neurons following acute administration. Indeed pre-treatment with either lithium or BDNF protected rat cerebral cortical neurons from glutamate excitotoxicity (Hashimoto et al., 2002), suggesting that BDNF is a key regulator of lithium-mediated neuroprotection. However, the therapeutic potential of BDNF is restricted, due to its relatively short half-life of less than 10 min, and inability to cross the blood-brain barrier (BBB; Wurzelmann et al., 2017). Small molecules that mimic BDNF’s function without its pharmacokinetic barriers would, therefore, be highly favorable. One such compound is 7,8-dihydroxyflavone (7,8-DHF), a selective TrkB agonist, which initiates activation of BDNF/TrkB signaling pathways, with a much longer half-life and greater permeability across the BBB than BDNF (Jang et al., 2010; Wurzelmann et al., 2017). Importantly, 7,8-DHF has been shown to have beneficial effects in cellular and animal models of AD, such as protecting primary neurons from Aβ-induced toxicity and promoting synaptogenesis (Zhang et al., 2014). Furthermore, chronic administration of 7,8-DHF prevented Aβ deposition, restored synaptic plasticity, and prevented memory deficits in AD transgenic mice (Zhang et al., 2014), suggesting that BDNF/TrkB signaling may have therapeutic potential in treatment of AD.

Glutamate is a major excitatory neurotransmitter in the CNS that plays important roles in synaptic plasticity and memory by initiating diverse signaling pathways including mTOR activation, phospholipase C, inositol triphosphate, ERK signaling and calcium release (Kumar et al., 2015; Ribeiro et al., 2017). Glutamate acts on ionotropic receptors such as *N*-methyl-D-asparate (NMDA), and metabotropic receptors to initiate an array of signaling responses (Kumar et al., 2015; Ribeiro et al., 2017). Metabotropic glutamate receptors (mGLuRs) are distributed throughout the CNS, and carry out multiple functions in maintaining synaptic transmission. Dysregulation of mGluRs have been implicated in several neurodegenerative diseases such as PD and AD, as well as diseases that affect intellectual capabilities including Fragile X Syndrome (Niswender and Conn, 2010; Ribeiro et al., 2017). Lithium attenuates the effects of hyperactive glutamate-mediated calcium signaling and has therefore been used as a therapy for conditions in which mGLuRs are dysfunctional (Sourial-Bassillious et al., 2009). For example, Fragile X mental retardation 1 (*Fmr1)* knockout mice have enhanced long term depression (LTD) in the hippocampus, due to increased activity of mGluR group I type 5 (Huber et al., 2002). Treating *Fmr1* knockout mice with lithium improved hyperactivity and rescued cognitive impairment by restoring mGluR-LTD (Choi et al., 2011; Liu et al., 2011). Similarly in *Drosophila* *fmr1* (*dfmr1)* mutant flies, lithium significantly improved mushroom body defects and restored learning and memory capabilities, as measured by altered courtship behavior, through a reduction in mGluR activity (McBride et al., 2005). Lithium affects signaling pathways that overlap with those regulated by mGluR. For example group 1 mGluRs mediate cerebellar mGluR-LTD by increasing inositol triphosphate (IP_3_) generation and releasing intracellular Ca^2+^ stores, and lithium is able to reverse these effects by inhibiting inositol levels (Berridge, 1993, 2009; Schloesser et al., 2008; Lüscher and Huber, 2010). Moreover, as previously mentioned, in lower model organisms loss of function mutations in the genes involved in inositol turnover, IMPA (*ttx* in *C. elegans*) and IPP (*ipp* in *Drosophila*), display defects in localization of synaptic components and synaptic transmission, respectively, which are phenocopied by lithium treatment (Acharya et al., 1998; Tanizawa et al., 2006). This strengthens the role of lithium in maintaining synaptic function by GSK-3 and IMPA-dependent mechanisms (see Figure 1B).

Although memantine, an NMDA antagonist, is currently used in symptomatic treatment of AD, long-term inhibition of this glutamate receptor may also lead to memory impairment due to its direct role in excitatory glutamatergic synaptic transmission. mGluR5 antagonists are therefore an attractive alternative, as mGluR5 plays a modulatory role in synaptic maintenance (Kumar et al., 2015). In particular, MPEP (2-Methyl-6-(phenylethynyl)pyridine) and MTEP(2-Methyl-4-thiazolyl)ethynyl)pyridine) are non-competitive mGluR antagonists that protect against neuronal toxicity *in vitro*, using primary neurons from AD transgenic mice, by preventing Aβ oligomer-induced dendritic spine loss (Um et al., 2013; Overk et al., 2014). Aβ oligomers interact with Prion protein (Prp^c^), and enhance the interaction between Prp^c^ and mGluR for signal transmission (Um et al., 2013; Haas et al., 2017). MPEP and MTEP inhibit both glutamate and Aβ/Prp^c^ signaling, while BMS-94923, a silent allosteric modulator does not alter glutamate signaling but rather inhibits the mGluR5-Prp^c^ interaction (Haas et al., 2017). Furthermore, BMS-94923 is able to prevent Aβ oligomer-induced inhibition of synaptic plasticity, and rescues memory deficits in a transgenic mouse model of AD, and as such this drug has great therapeutic potential for AD treatment, without the adverse side effects from modulating glutamate (Haas et al., 2017).

## Future Perspectives: Combination Therapies vs. Lithium Treatment for Dementia?

Lithium therefore has multi-factorial neuro-protective effects, and drugs targeting several of these mechanisms can slow cognitive decline in animal models of dementia (see Table 1). Combining such therapies may synergize these protective properties whilst excluding the toxic peripheral side effects, due to renal COX2 activation, observed with long-term lithium treatment. Although specific GSK-3 inhibitors, such as tideglusib, have reached clinical trial and appear to be safe for human use, they have so-far not shown significant therapeutic benefit in mild-moderate AD and further studies are required to optimize dosing and to measure effects at earlier disease stages (Lovestone et al., 2015). GSK-3 inhibition however does not recapitulate all of the protective features of lithium (see Figure 1), and this raises the possibility that their combined use with drugs targeting non-GSK-3-dependent mechanisms of lithium action may exert more effective neuro-protection under accumulating damage in dementia. Few studies have investigated these interactions, but additive protective effects have been observed when combining lithium with rapamycin or Keap1 inhibition in *Drosophila* models of HD and AD respectively (Sarkar et al., 2008; Kerr et al., 2017). Although lithium enhances autophagy by inhibiting IMPA, as described above, GSK-3 inhibition limits this protective property of lithium by activating mTOR. Combining lithium with the mTOR inhibitor rapamycin therefore enhances autophagy leading to improved efficacy against huntingtin-induced proteo-toxicity in comparison to treatment with either compound alone (Sarkar et al., 2008). Our studies also indicate that lithium, and TDZD-8, protect against Aβ42 neuro-toxicity predominantly through Nrf2-independent mechanisms and that dual Keap1-Nrf2 disruption and lithium treatment enhance neuro-protection through non-overlapping mechanisms (Kerr et al., 2017). Further work is required to uncouple these mechanisms using specific GSK-3 inhibitors and to optimize conditions for supplementing GSK-3 dependent neuroprotection with rapamycin and Nrf2 activators, as well as testing their combination with anti-inflammatory (TLR4 antagonists), neurogenic (TrKB agonists) and synaptic maintenance (mGluR antagonists) compounds, which have individual beneficial effects in neuroprotection (Table 1).

**Table 1:** Drugs targeting downstream mediators of lithium-mediated neuroprotection.

Class	Drugs	Neuroprotection *in vitro* and in animal models of neurodegeneration	Reference
GSK-3 inhibitors	ATP competitive: Indirubin Paullones Thiazoles Arylindolemaleimide (SB-216763 and SB-415286)	Neuroprotection, synaptic maintenance, improved cognitive function, reduced tau phosphorylation and, in some cases Aβ accumulation, in mouse models of AD and FTD.	Eldar-Finkelman and Martinez (2011)
	Non-ATP competitive: Thiadiazolidindiones (TDZD-8, NP12/tideglusib) L803-mts		
IMPA inhibitor	L-690,330	Enhanced degradation of mutant α-synuclein and huntingtin in PC12 neuroblastoma cells.	Sarkar et al. (2005)
Nrf2 activators	Triterpenoids (CDDO-MA, CDDO-EA, CDDO-TFEA)	Reduced oxidative stress, improved cognition and motor function in mouse models of AD and ALS.	Dumont et al. (2009), Neymotin et al. (2011)
	22h	Protected mouse primary neurons from amyloid-induced toxicity.	Kerr et al. (2017)
	Dimethyl fumarate (DMF)	Preserved viability against Aβ-induced toxicity in SHSY-5Y cell and mouse hippocampal slice cultures, and was neuro-protective in mouse models of PD and FTD.	Lastres-Becker et al. (2016), Campolo et al. (2018), Cuadrado et al. (2018)
BDNF/TrkB activator	dihydroxyflavone (7,8-DHF)	Protected primary neurons from Aβ-induced toxicity, and promoted synaptogenesis.	Zhang et al. (2014)
mGluR inhibitors	MPEP (2-Methyl-6-(phenylethynyl)pyridine), and MTEP(2-Methyl-4-thiazolyl)ethynyl)pyridine	Prevented toxicity in neuronal cultures and AD and DLB mouse models.	Um et al. (2013), Overk et al. (2014)
	BMS-94923	Prevented Aβ-induced inhibition of synaptic plasticity, and rescued memory deficits in an AD mouse model.	Haas et al. (2017)
TLR4 inhibitors	IAXO102 and FP7	Protected toxicity in primary neurons from the SOD1G93A mice model of ALS.	De Paola et al. (2016)
	Gx-50	Prevented Aβ-induced microglial activation and neuro-inflammation in rat microglia and APPswe, PSEN1dE9 mice.	Shi et al. (2016)

Alternatively, improved formulation and administration of lithium may better encapsulate the benefits of its neuroprotective properties in a single dementia therapy. Chronic (>15 months) microdose lithium administration has been suggested to stabilize memory impairment in AD patients (Nunes et al., 2013) and prevent neuropathology and cognitive decline in mice and rat models of AD with no observed toxicity (Nunes et al., 2015; Wilson et al., 2017). More recently LISPRO, a new ionic co-crystalized formulation of lithium salicylate and L-proline, has been reported to have improved brain penetrance and a safer pharmacokinetic profile over classical lithium chloride and carbonate salts (Habib et al., 2017). At therapeutically relevant doses, chronic LISPRO treatment prevented abnormal Aβ accumulation and tau phosphorylation, as well as neuronal and synaptic loss, in the Tg2576 mouse model of AD (Habib et al., 2017). Interestingly, this correlated with several of the established neuro-protective properties of lithium salts, including inhibition of GSK-3, enhanced autophagy, reduced neuro-inflammation and enhanced neurogenesis. Unlike lithium carbonate and lithium salicylate, however, acute or chronic LISPRO treatment did not enhance renal COX2 levels *in vitro* or *in vivo*, suggesting an improved safety profile by preventing the chronic renal toxicity observed using traditional lithium formulations.

The promise of lithium as a multi-functional therapy for the treatment of dementia therefore remains. Pre-clinical studies, across model organisms from flies to rodents, will prove vital however for systematic comparison of the effectiveness of combination treatments over new and microdose lithium formulations in slowing cognitive decline. Such analyses will guide the best approach for capturing the beneficial effects of lithium in the design of new disease-modifying therapies for clinical use in AD, as well as other neurodegenerative conditions for which lithium has shown favorable neuro-protective effects.

## Author Contributions

FK, IB and OS-A contributed equally to the conception, writing and editing of this review. FK and OS-A prepared the figures, tables and legends.

## Conflict of Interest Statement

The authors declare that the research was conducted in the absence of any commercial or financial relationships that could be construed as a potential conflict of interest.
